# Corneal Alterations Induced by Topical Application of Commercial Latanoprost, Travoprost and Bimatoprost in Rabbit

**DOI:** 10.1371/journal.pone.0089205

**Published:** 2014-03-14

**Authors:** Wensheng Chen, Nuo Dong, Caihong Huang, Zhenhao Zhang, Jiaoyue Hu, Hui Xie, Juxin Pan, Zuguo Liu

**Affiliations:** Eye Institute and Affiliated Xiamen Eye Center of Xiamen University, Fujian Provincial Key Laboratory of Ophthalmology and Vision Science, Fujian, China; Cedars-Sinai Medical Center; UCLA School of Medicine, United States of America

## Abstract

Prostaglandin (PG) analogs, including latanoprost, travoprost, and bimatoprost, are currently the most commonly used topical ocular hypotensive medications. The purpose of this study was to investigate the corneal alterations in rabbits following exposure to commercial solution of latanoprost, travoprost and bimatoprost. A total of 64 New Zealand albino rabbits were used and four groups of treatments were constituted. Commercial latanoprost, travoprost, bimatoprost or 0.02% benzalkonium chloride (BAK) was applied once daily to one eye each of rabbits for 30 days. The contralateral untreated eyes used as controls. Schirmer test, tear break-up time (BUT), rose Bengal and fluorescein staining were performed on days 5, 10, 20, and 30. Central corneal changes were analyzed by in vivo confocal microscopy, and the corneal barrier function was evaluated by measurement of corneal transepithelial electrical resistance on day 5. Whole mount corneas were analyzed by using fluorescence confocal microscopy for the presence of tight-junction (ZO-1, occludin) and adherens-junction (E-cadherin, β-catenin) proteins, actin cytoskeleton, proliferative marker Ki67 and cell apoptosis in the epithelium. Topical application of commercial PG analogs resulted in significant corneal epithelial and stromal defects while no significant changes in aqueous tear production, BUT, rose bengal and fluorescein staining scores on day 5. Commercial PG analogs induced dislocation of ZO-1 and occludin from their normal locus, disorganization of cortical actin cytoskeleton at the superficial layer, and disruption of epithelial barrier function. The eyes treated with 0.02% BAK and latanoprost exhibited significantly reduced Schirmer scores, BUT, and increased fluorescein staining scores on days 10 and 30, respectively. Topical application of commercial PG analogs can quickly impair the corneal epithelium and stroma without tear deficiency. Commercial PG analogs break down the barrier integrity of corneal epithelium, concomitant with the disruption of cell junction and actin cytoskeleton between superficial cells in the corneal epithelium *in vivo*.

## Introduction

The corneal epithelium provides a physical and functional barrier that protects the eye from harmful external agents, such as microbes and chemicals, and contributes to the transparency of the cornea [1]–[3]. Tight junctions (TJs) between adjacent epithelial cells at the apical plasma membrane play a crucial role in the establishment and maintenance of the barrier function and cell polarity [4]–[7]. TJs are composed of zonula occludens (ZO)-1, ZO-2, ZO-3, occluden, claudin, and junctional adhesion molecules, which interact with their counterparts in the neighboring cells. These interactions are stabilized by intercellular tethering forces generated by the adherens junctions (AJs), which are proximal to TJs. The protein components of AJs include cadherins and catenins (α-catenin, β-catenin, p120 catenin). The cytoplasmic domains of the transmembrane molecules of TJs and of the AJs are structurally and functionally linked to the actin cytoskeleton via adapter molecules, such as ZO-1, ZO-2, and β-catenin. These cell junctional proteins have been shown to be important for corneal epithelial barrier function[8].

Glaucoma is the second leading cause of blindness in the world. Among the current ocular hypotensive medications employed in the treatment of open-angle glaucoma and ocular hypertension, the first-line choice is topical application of prostaglandin (PG) analogs based upon their efficacy in lowering intraocular pressure, few systemic side effects and ease of once-daily dosing [9], [10]. However, clinical studies have shown that topical application of PG analogs can cause ocular surface toxicity [11], [12].

The toxicity of commercial PG analogs has often been associated with the most common preservative benzalkonium chloride (BAK). Recently, using an acute toxicological model, Liang et al. found that BAK-preserved PG analogs exhibited higher proinflammatory effects on the ocular surface [13]. Nakagawa et al. compared the effects of BAK-preserved and unpreserved antiglaucoma topical eye drops on the human 3D-reconstituted corneal epithelial model [14]. They found that ZO-1 protein expression was significantly decreased following exposure to BAK-preserved latanoprost [14]. However, *in vivo* effect of commercial PG analogs on the barrier function of the corneal epithelium remains largely unclear.

We have previously proven that topical application of BAK disrupts the TJs of corneal epithelium between superficial cells in the corneal epithelium *in vivo* [15], [16]. In the present study, we evaluated the corneal alterations induced by commercial latanoprost, travoprost and bimatoprost in rabbits. We were particularly interested in investigating the *in vivo* effect of these PG analogs on the barrier function of the corneal epithelium.

## Methods

### Animal and antiglaucoma eye drop treatment

All experiments were conducted in accordance with the ARVO Statement for Use of Animals in Ophthalmic and Vision Research and approved by the animal ethics committee of Xiamen University School of Medicine (approval ID: XMUMC: 2013-01-29). Sixty-four male New Zealand rabbits (obtained from Shanghai Medical Laborarory Animal center, Shanghai, China) weighing between 2 and 2.5 kg were randomly assigned to four groups of 16 rabbits each. The animals were screened for ocular surface disease with a handheld biomicroscope before any experimental procedure and were excluded if any disease was found. Commercial 0.005% latanoprost containing 0.02% BAK (Xalatan®; Pfizer, New York, NY), 0.04% travoprost containing 0.015% BAK (Travatan®; Alcon, Fort Worth, TX), 0.003% bimatoprost containing 0.005% BAK (Lumigan®; Allergan, Irvrine, CA) or 0.02% BAK was applied to one eye of each rabbit once daily (9 AM) for 30 days, with the second eye of each animal serving as normal control. Clinical evaluations were performed following the methods described below on days 0, 5, 10, 20, and 30.

### Clinical Ocular Surface Evaluations

Each rabbit underwent a clinical ocular surface examination, including aqueous tear production, fluorescein staining and tear break-up time (BUT).

Tear Production: Tear production was measured with Schirmer paper strips (Tianjin Jingming New Technology Development Co., Ltd, Tianjin China). The strip was placed in the outer third of the lower eyelid for 5 minutes without anesthesia. The length of the moistened portion of the strip was recorded to an accuracy of 0.5 mm. The test was repeated three times, and then the mean value was obtained.

Corneal Fluorescein Staining and BUT: Two μl of 1% sodium fluorescein was instilled into the lower fornix of the conjunctiva. The rabbit was allowed to blink several times to distribute the fluorescein evenly on the cornea. The time from opening of the eyes to the appearance of the first dry spot in the central cornea was measured 3 times and the mean was recorded with a cobalt blue filter under a slit-lamp microscope (BQ900® Haag-Streit, Bern, Switzerland). Two minutes later, corneal fluorescein staining intensity was also examined and graded under the slit-lamp microscopy.

### *In Vivo* Confocal Microscopy Observation

On day 5, the rabbits were injected intraperitoneallly with a mixture of xylazine (1 mg/kg body weight; Bayer, Shawnee Mission, KS) and sodium pentobarbital (20 mg/kg; Abbott Laboratories, North Chicago, IL) to keep the animals immobile. After applying a drop of carmober gel (Alcon Laboratories, Fort Worth, TX), a laser scanning *in vivo* confocal microscopy Heideberg Retina Tomograph III/Rostock Cornea Module (Heidelberg Engineer GmbH, Heideberg, Germany) was used to examine the central cornea of the anesthetized rabbits, including superficial epithelium, stroma and endothelium, as previously described [15], [16]. The mean central corneal thickness was calculated based on the depth difference between the most superficial epithelium and endothelium and was recorded as the average of a minimum of three individual acquisitions. At the end, the epithelial superficial cell size and endothelial cell density were automatically calculated using the tomography-associated software. Based on 10 images, the means and standard deviations were calculated for each parameter.

### Measurement of Corneal Transepithelial Electrical Resistance (TER)

Corneal TER was measured as previously described [16]. Briefly, a 1.0-mm-diameter Ag/AgCl electrode (Physiotech, Tokyo, Japan) was inserted into the anterior chamber through a small incision in the peripheral cornea, which had been made with an 18-gauge sharp needle (Terumo, Tokyo, Japan). Using biomedical adhesive (Alon-Alpha A; Sankyo), a 6.0-mm-internal diameter (0.28-cm^2^ inner area) nitrile rubber O-ring (Union Packing; SAN-EI, Osaka, Japan) was fixed on the cornea. Then, 60 μL of HBSS was placed inside the ring at the center of the cornea, and the other electrode was carefully placed on the cornea. The TER was measured using a volt-ohm meter (EVOMX, World precision Instruments, Saeasota, FL), which generates ±20-μA alternating current (AC) square wave current at 12.5 Hz, and data were recorded using a thermal arraycorder (WR 300-8; Graphtech, Tokyo, Japan).

### Immunofluoresecence Analysis

Rabbits were killed with a lethal dose of pentobarbital sodium (100 mg/kg) injected intravenously. After the eyes had been enucleated and fixed in PBS with 4% paraformaldehyde for 3 minutes, under a dissecting microscope (Model SZ40; Olympus, Tokyo, Japan), the retina, lens and iris were discarded, and four incisions were made in each cornea. Subsequently, the corneas were permeabilized with acetone for 3 minutes at −20°C. After washing in PBS with 1% Triton X-100 and 1% dimethyl sulfoxide (DMSO; TD buffer), the tissue blocks were incubated in 1% BSA diluted in TD buffer for 1 hour to block nonspecific binding. Then, the tissue blocks were incubated overnight at 4°C with mouse antibodies to ZO-1 (Zymed, Carlabad, CA), occludin (Zymed, Carlabad, CA), E-cadherin (BD, Biosciences, Carlabad, CA) or β-actenin (BD, Biosciences, Carlabad, CA), each at a 1∶100 dilution in TD buffer containing 1% BSA. The next day, after washing with TD buffer, the tissue blocks were incubated at 4°C for 1 hour with AlexaFluor 488-labeled secondary antibody (Molecular Probes; Eugene, OR) at a 1∶1000 dilution in TD buffer. Afterward, the tissues were incubated in anti-Ki67 antibody (1∶100; Abcam) for 8 h with agitation. The tissues were then rinsed with TD buffer and placed in Alexa Fluor 594-conjugated secondary antibody for 2 h with agitation. After distilled water wash, the whole-mount cornea tissues were mounted epithelial side up on a slide and stained with a nuclear fluorescence dye, 4,6-diamidino-2-phenylindole (DAPI, Vector, Laboratories, Burlingame CA). For actin cytoskeleton staining, the whole mount corneas were incubated in 1∶1000 of Texas-red conjugated phalloidin (Molecular Prbes, Eugene, OR) overnight with agitation for 4 hours at 4°C. The tissues were rinsed three times in PBS (5 minutes per rinse), and then the whole mount epithelial side up on a slide and stained with DAPI.

To measure end-stage apoptosis, in situ TUNEL was performed in whole mount corneas using a TUNEL apoptosis detection kit (DeadEnd Fluorometric TUNEL, System G3250; Promega, Madison, WI) according to manufacturer' instructions. After distilled water wash, the corneal tissues were mounted and stained with DAPI.

The corneal tissues were examined with a laser confocal microscopy (Olympus Fluoview 1000; Olympus, Japan). All the corneal tissues that had been observed on whole mount were snap-frozen in liquid nitrogen, and 8 μm thick cross-sections were prepared with cryostat. These sections were also examined by the confocal microscopy.

### Statistical Analysis

Quantitative data are expressed as mean ± SD from three independent experiments and were analyzed by Dunnett multiple comparison test. P<0.05 was considered statistically significant.

## Results

### Clinical Findings

**Figure 1:**
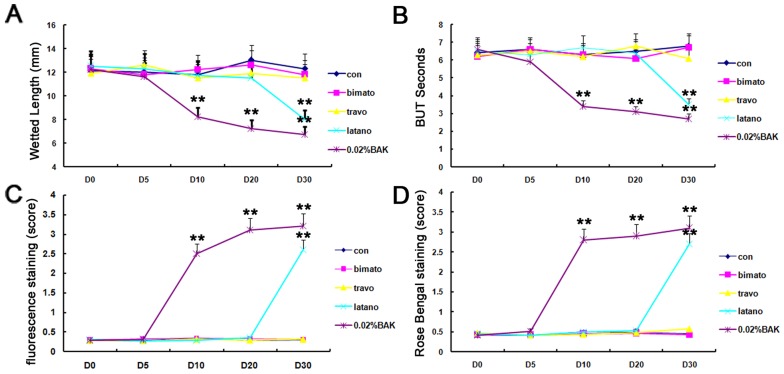
Alterations of the clinical evaluations of the ocular surface on days 0, 5, 10, 20 and 30 in each group. (A) Schirmer test. (B) Fluorescein score. (C) BUT. (D) Rose Bengal score. The travopost and bimatoprost groups showed no statistical differences in aqueous tear production, BUT, corneal fluorescein and rose bengal scores compared with the control group at each time point. In contrast, the eyes treated with 0.02% BAK and latanoprost showed significant increases in the fleorescein and rose Bengal scores, and decreases in Schirmer score and BUT on day 10 and 30, respectively. Data show mean ± SD of values from four eyes per group. **P<0.01.

The travopost and bimatoprost groups showed no statistical differences in aqueous tear production, BUT, corneal fluorescein and rose bengal scores compared with the control group at each time point. In contrast, 0.02% BAK and latanoprost resulted in significant increases in the corneal fluorescein and rose Bengal staining scores, decreases in BUT, and aqueous tear production on days 10 and 30, respectively (Fig. 1).

### *In Vivo* confocal Microscopy Analysis

**Figure 2:**
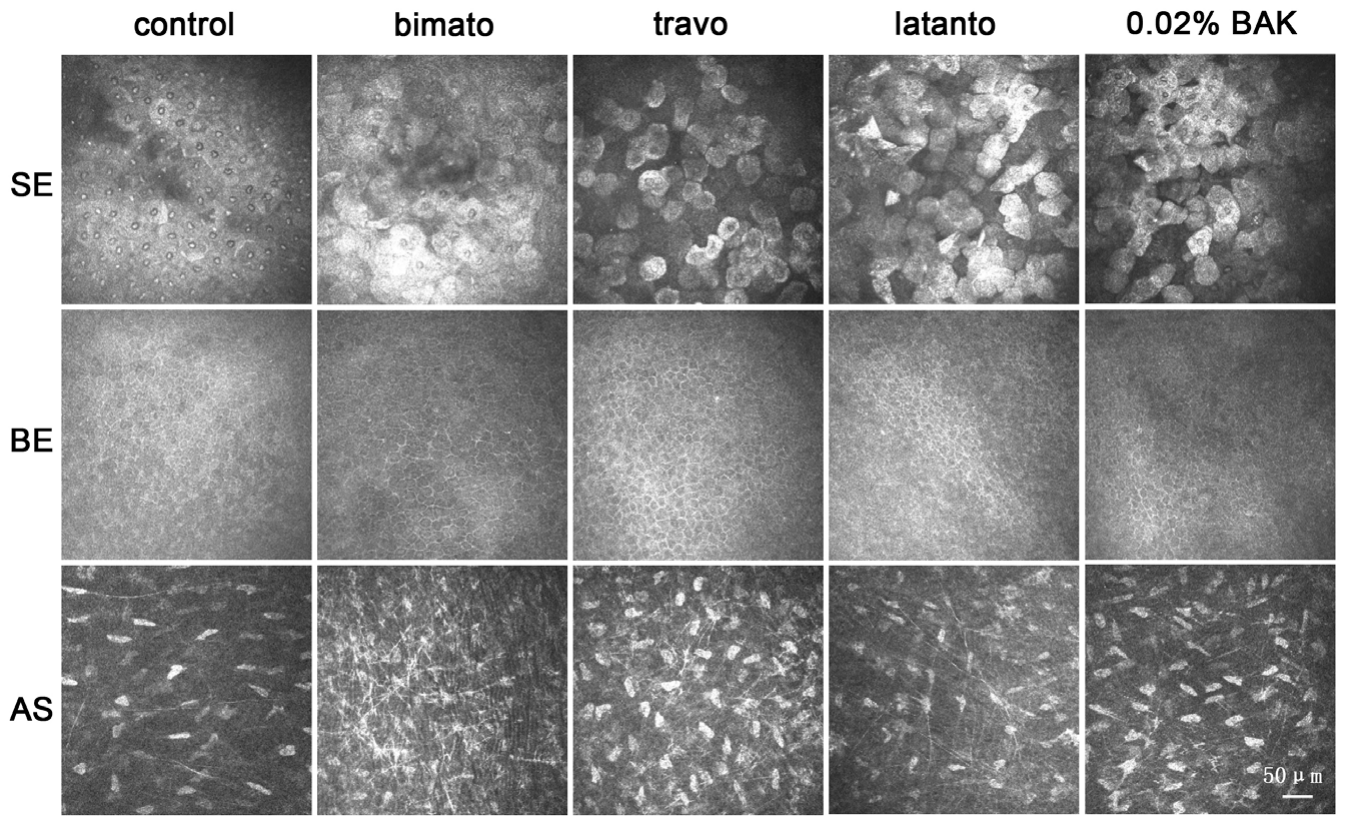
Representative *in vivo* confocal microscopic images showing corneal alteration with commercial PG analogs and BAK treatment on 5 day. The images are representative of at least three independent experiments. Note that surface cells in the corneal epithelium of the eyes treated with commercial PG analogs and BAK were larger, with blurry boundaries and abnormal reflectivity, compared with those of control eyes. In the eyes treated with bimatoprost, a large number of membrane bridge-like structures were present in the anterior stroma. SE: superficial epithelium. BE: basal epithelium. AS: anterior stroma.

**Figure 3:**
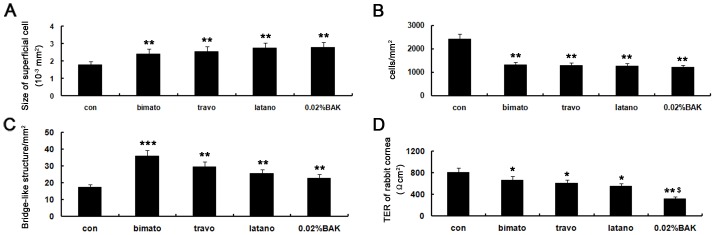
Representative images showing the size of corneal superficial epithelial cells, corneal epithelial superficial cell density, mean corneal anterior stromal membrane bridge-like structure, and corneal TER with commercial PG analogs and BAK treatment on day 5. The size of surface cells in the corneal epithelium of eyes treated with commercial PG analogs and BAK was significantly larger than that of control eyes (A). The corneal superficial cell density (B) was significantly decreased and significantly higher densities of membrane bridge-like structures (C) in the corneal anterior stroma were indentified in PG analogs and BAK treated corneas. PG analogs induced a significant decrease in corneal TER. BAK at 0.02% considerably decreased corneal TER, with statistically significant difference compared with commercial latanoprost (D). Data show mean ± SD of values from four eyes per group. *P<0.05, **P<0.01, ***P<0.001(Dunnett test). $: Statistically significant difference compared with latanoprost (*P*<0.05).

The control rabbits presented normal aspects of the corneal superficial epithelium, with regular polygonal mosaic appearance, brightly reflective nuclei and no obvious desquamation or swelling. The commercial PG analogs and BAK treated eyes displayed various abnormalities of the corneal superficial epithelial cells, including partial desquamation of epithelial cells, irregular cell shapes, anisocytosis and loss cell borders, brightly reflective round mosaic appearance, swollen (Fig. 2). The sizes of the corneal superficial epithelial cells of eyes treated with bimatoprost, travoprost, latanoprost and 0.02% BAK were significantly larger, by 35.2%, 42.3% and 52.6%, 55.2% than that of control eyes, respectively (Fig. 3A, B).

The control basal epithelial cells appear as regular mosaic of dark cell bodies with light, narrow inter-cellular borders. No significant inflammatory infiltration in basal epithelium was observed with any treatment (Fig. 2).

In control central anterior stroma, hyperreflective membrane bridge-like structures were occasionally observed interconnecting two or more cells (Fig. 2). These highly distinctive intercellular membrane bridge-like structures were generally straight, single, fine (<0.5 μm diameter), long (>60 μm) and less ramified. Membrane bridge-like structures in the anterior stroma of the eyes treated with bimatoprost and travoprost were distributed in a net-like formation (Fig. 2). The numbers of these structures were increased by 108%, 72%, 48% and 32% in the eyes treated with bimatoprost, travoprost, latanoprost and 0.02% BAK, respectively (Fig. 3 C).

The morphology of posterior stroma and endothelium did not appear obvious differences between control and treated eyes. In addition, there was no significant difference between the control and treated eyes in central corneal thickness.

### Effect of commercial latanoprost, travoprost, bimatoprost and BAK on corneal TER

To assess the effect of commercial latanoprost, travoprost and bimatoprost on corneal barrier function, we measured the corneal TER of living rabbits on day 5 (n = 4 in each group). As shown in Fig.3 D, exposure of the cornea to commercial PG analogs resulted in a significant decrease in TER in BAK-dependent manner. BAK at 0.02% considerably decreased corneal TER, with statistically significant difference compared with commercial latanoprost.

### Effect of Commercial Latanoprost, Travoprost, Bimatoprost and BAK on the Localization of TJs and AJs Proteins in the Rabbit Corneal Epithelium

**Figure 4:**
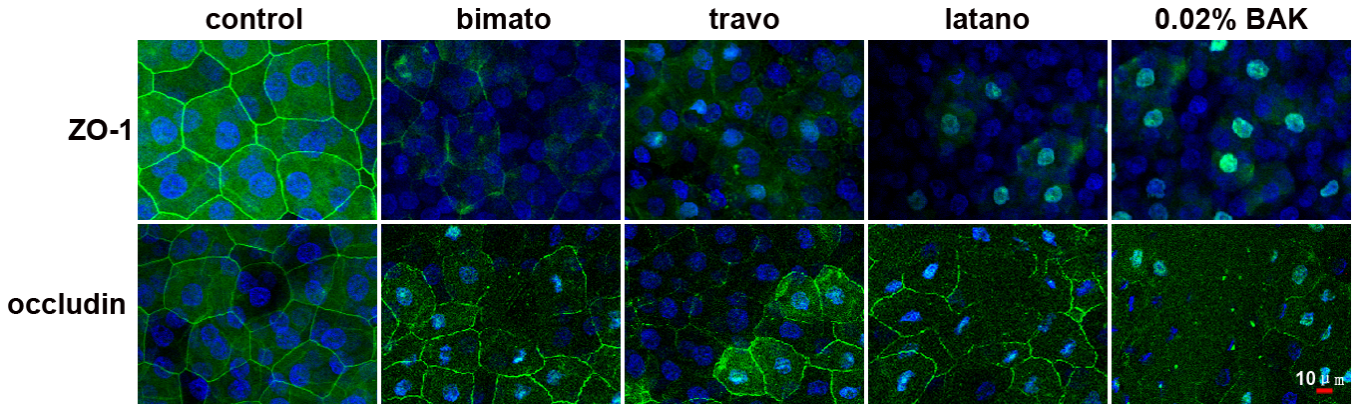
Representative images showing the distribution of ZO-1 and occludin in the rabbit corneal epithelium with commercial PG analogs and BAK treatment on day 5. Corneal tissue blocks prepared from a control eye or from eyes treated with bimatoprost, travoprost, latanoprost or 0.02% BAK. The images are representative of at least three independent experiments. ZO-1 and occludin staining was observed as a continuous linear pattern along with superficial cell-cell borders in normal rabbit corneal epithelial cells. ZO-1 and occludin staining was discontinuous in the eyes treated with commercial PG analogs and 0.02% BAK.

**Figure 5:**
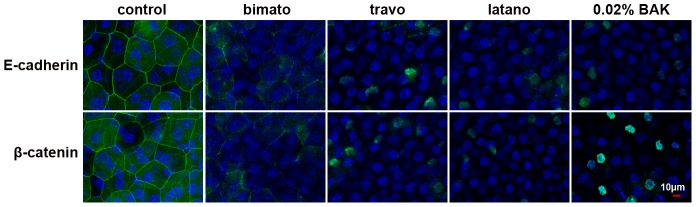
Representative images showing the cortical actin cytoskeleton of the rabbit corneal epithelial cells with commercial PG analogs and BAK treatment on day 5. Rabbit corneas were fixed and stained with Texas red phalloidin. The images are representative of at least three independent experiments. Exposure to commercial PG analogs and BAK caused disruption of the cortical actin bundles of the corneal epithelial superficial cells. In contrast, the pattern of the cortical actin cytoskeleton of the corneal epithelial basal cells of the eyes treated with commercial PG analogs and BAK was similar to that of untreated eyes. SE: superficial epithelium, BE: basal epithelium.

To investigate the in vivo effect of commercial latanoprost, travoprost and bimatoprost on the components of TJs and AJs-associated proteins, immunofluorescent staining was performed on days 5 and 30 (n = 4 in each group). In control corneal epithelia, ZO-1, occludin, E-cadherin and β-catenin were all localized contiguously at the superficial cell-cell boundaries and accordingly stained at the cell borders. Cross-sectional studies further confirmed that ZO-1, occludin, E-cadherin and β-catenin were all exclusively located in the corneal superficial epithelial layer (data not shown). Commercial PG analogs and 0.02% BAK treatment resulted in a loss of ZO-1 and occludin from the superficial epithelial cellular border (Fig. 4), but it did not affect the distribution of E-cadherin and β-catenin on day 5 (data not shown). Commercial PG induced the disruption of tight junction as well as dispersion of E-cadherin and β-catenin at the superficial layer of the corneal epithelium on day 30 (Fig. 5). These observations thus suggested that exposure to commercial PG analogs could disrupt the localization of both AJ and TJ proteins.

### Effect of Commercial Latanoprost, Travoprost, Bimatoprost and BAK on Actin Cytoskeleton

**Figure 6:**
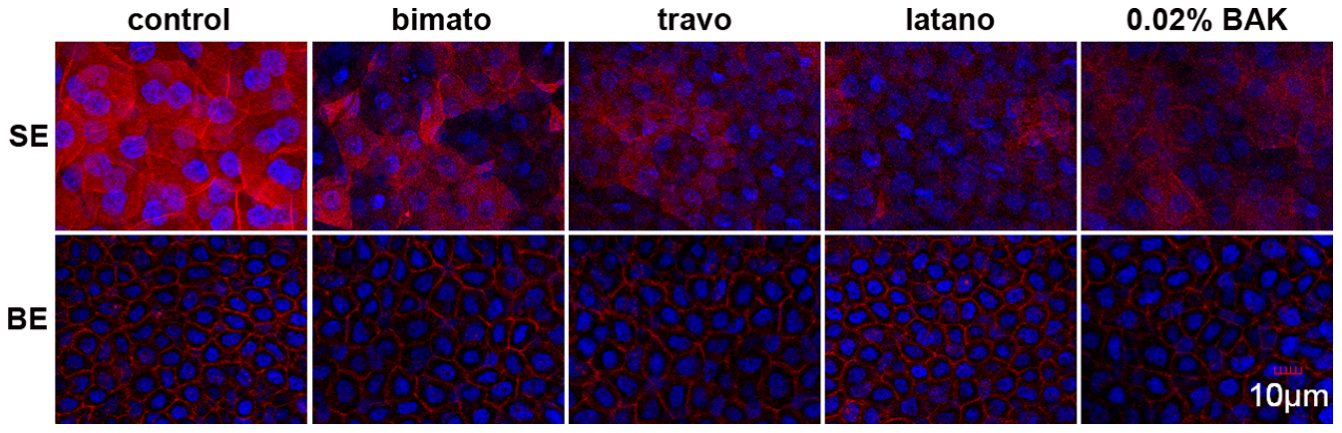
Representative images showing the distribution of E-cadherin and β-catenin in the rabbit corneal epithelium with commercial PG analogs and BAK treatment on day 30. Corneal tissue blocks prepared from a control eye or from eyes treated with bimatoprost, travoprost, latanoprost or 0.02% BAK. The images are representative of at least three independent experiments. E-cadherin and β-catenin staining was observed as a continuous linear pattern along with superficial cell-cell borders in normal rabbit corneal epithelial cells. E-cadherin and β-catenin staining was discontinuous in the eyes treated with commercial PG analogs and 0.02% BAK.

We examined organization of the actin cytoskeleton in response to commercial latanoprost, travoprost, bimatoprost and 0.02% BAK *in vivo* using phalloidin (conjugated to Texas Red) staining on days 5 and 30 (n = 4 in each group). The control corneal epithelial cells showed a dense web of cortical actin. At each time point, F-actin staining was patchy in the corneal superficial epithelium of the eyes treated with commercial PG analogs and BAK. This change was more prominent in the eyes treated with 0.02% BAK. In the basal epithelium, however, the pattern of F-actin staining distribution is similar to that of control (Fig. 6). These observations thus suggested that commercial PG analogs induced disruption of the actin cytoskeleton in the corneal superficial epithelium.

### Effect of Commercial Latanoprost, Travoprost, Bimatoprost and BAK on Corneal Epithelial Cell Proliferation and Apoptosis

**Figure 7:**
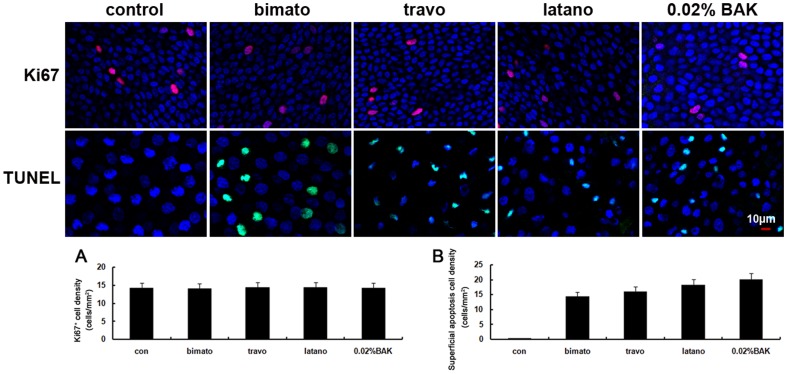
Representative images showing the cell proliferation and apoptosis of the rabbit corneal epithelial cells with commercial PG analogs and BAK treatment on day 5. Corneal tissue blocks prepared from a control eye or from eyes treated with bimatoprost, travoprost, latanoprost and 0.02% BAK. The images are representative of at least three independent experiments. Representative images for Ki67 staining and TUNEL assay were shown. The expression of Ki67 was shown in (A). Mean superficial apoptotic cells were shown in (B). Data show mean ± SD of values from four eyes per group.

To characterize the proliferation status of the corneal epithelial cells following exposure to commercial PG analogs, we performed immunofluorescence staining for Ki67. Immunofluoresence staining of control corneal tissues with Ki67 antibody revealed that Ki67 positive cells were exclusively located in the basal layer (Fig. 7). Commercial PG analogs and 0.02% BAK did not induced significant changes of the number of Ki67 positive cells in the basal layer on day 5.

We also performed a TUNEL assay to detect apoptosis of corneal epithelial cells, and the results showed that no apoptotic cells were present in the control corneas. Commercial PG analogs and 0.02% BAK induced apoptosis in the superficial epithelium on day 5. The apoptotic cells were counted, giving densities of 13.2±2.8, 16.5±3.6, 19.6±5.5 and 22.6±3.8 cells/mm^2^ in the eyes treated with latanoprost, travoprost, bimatoprost and 0.02% BAK, respectively (Fig. 7).

## Discussion

PG analogs are the most frequently used topical ocular hypotensive medications for their apparently good safety/efficacy profile [11]. However, the generally good hypotensive outcomes have tended to overshadow the significant number of patients who undergo ocular surface discomfort. Therefore, prophylaxis and treatment of ocular surface toxic reactions associated with commercial PG analogs have become important clinical issues. In the present study, we utilized a clinically relevant animal model to examine the corneal alterations following exposure to commercial solution of latanoprost, travoprost and bimatoprost.

We have now shown that optical application of commercial PG analogs can quickly break down the barrier integrity of corneal epithelium without causing significant changes in aqueous tear production, corneal fluorescein and Rose bengal scores, and BUT. The findings indicated that the barrier function of corneal epithelium is very sensitive to the toxicity of commercial PG analogs. Kusano et al [17] recently developed an in vivo corneal TER measurement system that can be used to measure the barrier function of the intact fresh cornea in live rabbits. Nakagawa et al [14] reported that exposure to commercial PG analogs induced the disruption of the barrier function of stratified cultivated human corneal epithelial cell sheets. Our study extends these findings and confirms that *in vivo* toxic effect of commercial PG analogs on corneal epithelial barrier function is dependent on BAK concentrations. We found that commercial latanoprost (containing 0.02% BAK) has less toxic effects on the barrier function than 0.02% BAK. In conjunctival cell culture, the active component of PG analogs produced a marked reduction of various inflammatory cytokines [18], [19], and were responsible for protective effects against BAK toxicity by their antioxidative properties [20]. We data suggest possible protective effect of the active component latanoprost on corneal epithelium from barrier function disruption induced by BAK.

Corneal epithelium serves as a functional barrier between the external and internal ocular environment. Both tight and adherens junctions contribute to the establishment and maintenance of this barrier. ZO-1 is expressed in the superficial cell layer of the corneal epithelium and has been considered a marker of the tight junction [16], [21]. Recently, we found that topical application of BAK results in the redistribution of ZO-1 and in the disruption of the barrier function of corneal epithelium [15], [16]. In this study, we found that control eyes exhibited a continuous linear pattern of tight junction proteins staining at cell-cell boundaries in the surface of the rabbit corneal epithelium *in vivo*. In the eyes treated with commercial PG analogs, ZO-1 and occludin immunoreactivity was discontinuous, suggestive of disruption of tight junction. We noted that commercial PG analogs induced dispersion of ZO-1 and occludin from the interfaces of neighboring corneal superficial epithelial cells without affecting the localization of E-cadherin and β-catenin on day 5. These findings indicate that topical application of commercial PG analogs can disrupt the barrier integrity of the cornea as a consequence of dispersion of TJ proteins from their normal locus at superficial epithelial layer. We also noted that commercial PG analogs induced dispersion of E-cadherin and β-catenin at the superficial layer of the corneal epithelium on day 30. These findings suggested that the long-term use of commercial PG analogs induces the disruption of AJs between superficial cells in the rabbit corneal epithelium *in vivo*.

Both tight and adherens junctions are associated with the actin cytoskeleton, which plays an important role in the development and maturation of intercellular adhesion. It has been demonstrated that BAK can induce contraction of cortical actin filaments at junctional structures, which results in disruption of corneal epithelial cell barrier function [22]. In this study, we found that in commercial PG analogs treated eyes, F-actin immunoreactivity was patchy in the superficial epithelium. Our study has demonstrated that commercial PG analogs break down the barrier integrity of the corneal epithelium, concomitant with the disruption of cell junction and actin cytoskeleton between superficial cells in the rabbit corneal epithelium *in vivo*.

Chinnery et al. reported that the corneal stroma of normal mouse is endowed with a small number of long (>300 μm) and fine (<0.8 μm) membrane nanotube-like structures with expression of histocompatibility complex class II molecules [23]. They showed that the frequency of these nanotubes was significantly increased in corneas subjected to trauma and LPS, suggesting that these structures play an important role *in vivo* in cell-cell communication between widely spaced dendritic cells during inflammation [23]. We have previously demonstrated existence of long and hypereflective membrane bridge-like structures in intact rabbit corneal anterior stroma [15]. In this study, we noted that 5 days after application of commercial bimatoprost, the number of membrane bridge-like structures in central anterior stroma increased from 17.2±1.5 per square millimeter to 35±6.6 per square millimeter. This finding suggests that these membrane bridge-like structures have an important role *in vivo* in commercial bimatoprost induced corneal stromal inflammation. We also noted that the density of these structures was less increased in the eyes treated with commercial travoprost, latanoprost and 0.02% BAK. Guenoun et al. investigated the effect of commercial PG analogs on conjunctiva-derived epithelial cells [18]. They found that although bimatoprost contains less BAK that do travoprost and latanoprost, the expression levels of various inflammatory cytokines were higher than those obtained with two other PG analogs [18]. Our results support this observation.

We investigated the effect of commercial PG analogs on cell proliferation and apoptosis of rabbit corneal epithelium. It has been shown that in 3D-reconstituted corneal epithelium, the number of apoptotic and proliferative cells increased following exposure to BAK containing commercial PG analogs [24]. Our study provides direct evidence that commercial PG analogs can induce corneal epithelial cell apoptosis in a BAK-concentration-dependent manner without affecting cell proliferation *in vivo*.

A significant difference between the commercial PG analogs is the concentration of the preservative BAK. BAK can affect the tear film *in vivo* due to its detergent effect [25]. In this study, we found that after commercial PG analogs treatment dry eye symptoms occurred only in the eyes treated with latanoprost, which contains the highest concentration of BAK. This finding is agreement with a recent study reporting significant decreases in Schirmer score and goblet cell density and increase in fluorescein score in rabbits after 30 days treatment of commercial latanoprost [26]. Our group has proven that the reduced aqueous tear production in eyes treated with BAK is a consequence of corneal nerve damage [27]. Further studies are needed to determine the effect of commercial PG analogs on corneal nerves.
