# Correction: Corneal Alterations Induced by Topical Application of Commercial Latanoprost, Travoprost and Bimatoprost in Rabbit

**DOI:** 10.1371/journal.pone.0098378

**Published:** 2014-05-16

**Authors:** The PLOS ONE Staff

There is an error in Figure 4. One panel (staining for occludin by latano) is an accidental duplication of Figure 7C in the authors’ previously published paper [2] with different magnification. The authors have provided a corrected Figure 4 here.

**Figure 4:**
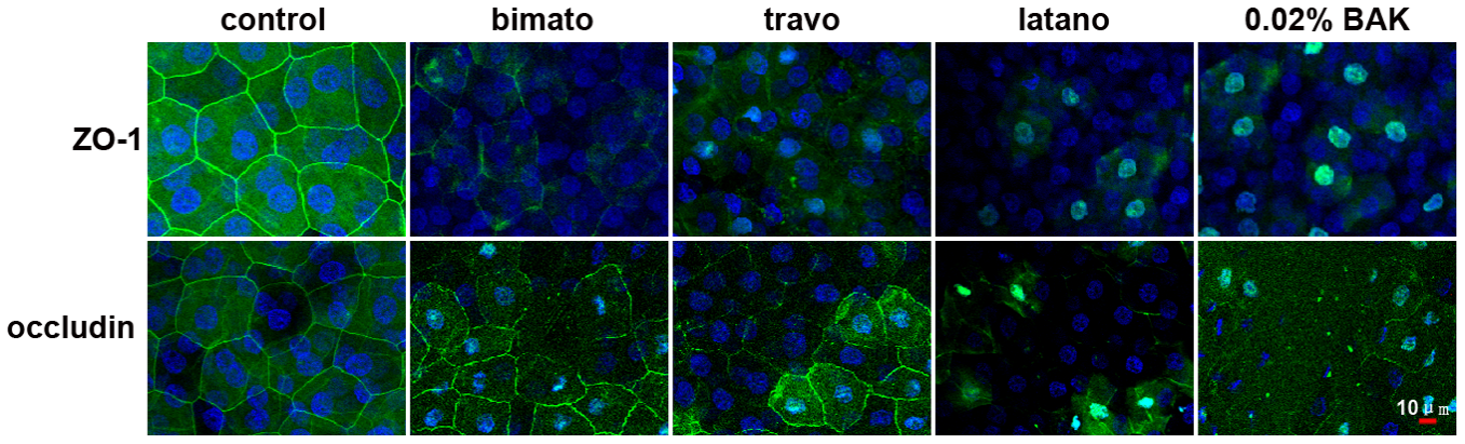
Representative images showing the distribution of ZO-1 and occludin in the rabbit corneal epithelium with commercial PG analogs and BAK treatment on day 5. Corneal tissue blocks prepared from a control eye or from eyes treated with bimatoprost, travoprost, latanoprost or 0.02% BAK. The images are representative of at least three independent experiments. ZO-1 and occludin staining was observed as a continuous linear pattern along with superficial cell-cell borders in normal rabbit corneal epithelial cells. ZO-1 and occludin staining was discontinuous in the eyes treated with commercial PG analogs and 0.02% BAK

In addition, the legends of Figure 5 and Figure 6 are switched. Please see the corrected legends here.

**Figure 5:**
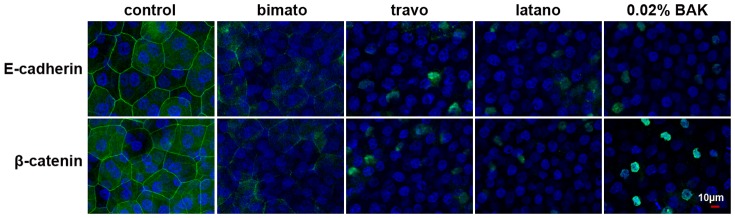
Representative images showing the distribution of E-cadherin and β-catenin in the rabbit corneal epithelium with commercial PG analogs and BAK treatment on day 30. Corneal tissue blocks prepared from a control eye or from eyes treated with bimatoprost, travoprost, latanoprost or 0.02% BAK. The images are representative of at least three independent experiments. E-cadherin and β-catenin staining was observed as a continuous linear pattern along with superficial cell-cell borders in normal rabbit corneal epithelial cells. E-cadherin and β-catenin staining was discontinuous in the eyes treated with commercial PG analogs and 0.02% BAK.

**Figure 6:**
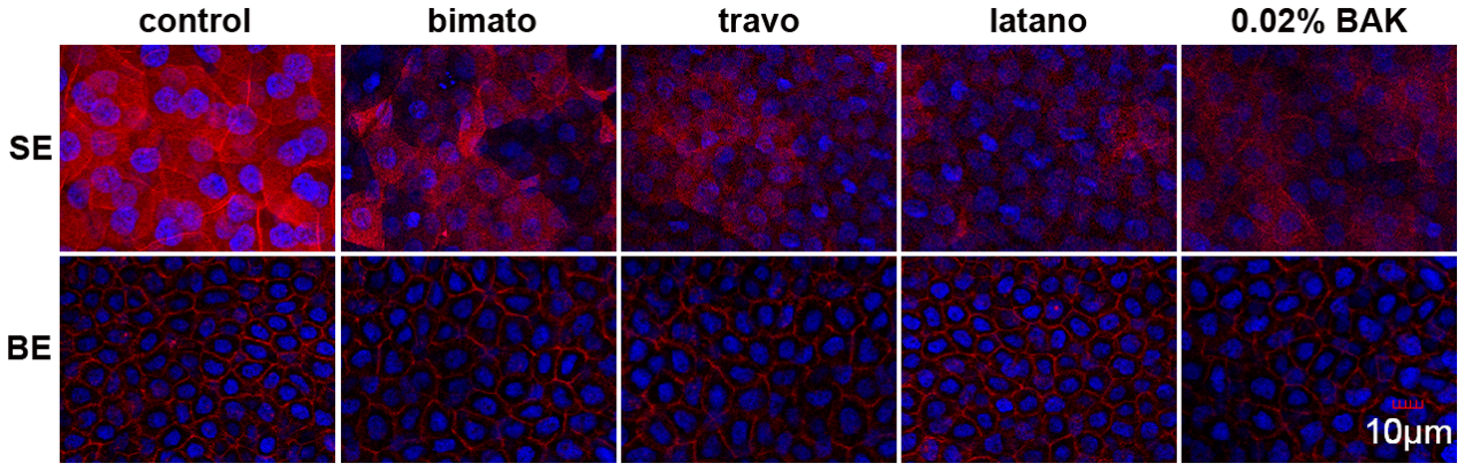
Representative images showing the cortical actin cytoskeleton of the rabbit corneal epithelial cells with commercial PG analogs and BAK treatment on day 5. Rabbit corneas were fixed and stained with Texas red phalloidin. The images are representative of at least three independent experiments. Exposure to commercial PG analogs and BAK caused disruption of the cortical actin bundles of the corneal epithelial superficial cells. In contrast, the pattern of the cortical actin cytoskeleton of the corneal epithelial basal cells of the eyes treated with commercial PG analogs and BAK was similar to that of untreated eyes. SE: superficial epithelium, BE: basal epithelium.
